# Severe acute respiratory syndrome coronavirus-2 in post-laryngectomy patients: case series of four patients

**DOI:** 10.1017/S0022215120001310

**Published:** 2020-06-23

**Authors:** H Coleman, J Sutherland, N Calder

**Affiliations:** ENT Department, University Hospital Monklands, Airdrie, Scotland, UK

**Keywords:** Coronavirus, COVID-19, Laryngectomy, Squamous Cell Carcinoma

## Abstract

**Objective:**

To report our experience of diagnosis, investigation and management in patients who had undergone laryngectomy secondary to previous squamous cell carcinoma, who were subsequently infected with severe acute respiratory syndrome coronavirus-2 during the coronavirus disease 2019 pandemic.

**Case reports:**

Four post-laryngectomy patients with laboratory-proven severe acute respiratory syndrome coronavirus-2 infection were admitted to our institution from 1 March to 1 May 2020. All patients displayed symptoms of coronavirus disease 2019 and underwent investigations, including swab and serum sampling, and chest X-ray where indicated. All were managed conservatively on dedicated coronavirus disease 2019 wards and were discharged without the requirement of higher level care.

**Conclusion:**

It is hypothesised that laryngectomy may offer a protective effect against severe or critical disease in severe acute respiratory syndrome coronavirus-2 infection. We hope sharing our experience will aid all practitioners in the management of this, often intimidating, cohort of patients.

## Introduction

In December 2019, cases of what appeared to be an acute atypical pneumonia began to cluster in Wuhan, China. Rapid spread to surrounding areas lead to the discovery of a novel coronavirus now known as severe acute respiratory syndrome coronavirus-2 (SARS-CoV-2), resulting in the disease known as coronavirus disease 2019 (Covid-19). The World Health Organization declared Covid-19 a global pandemic on 11 March 2020, and, as of 25 April 2020, there have been 2 800 000 cases and 190 000 deaths worldwide.^1^

Surgically managed head and neck cancer patients are thought to be at an increased risk of SARS-CoV-2 infection. Having undergone total laryngectomy and possible adjuvant treatment such as chemotherapy and/or radiotherapy for squamous cell carcinoma (SCC), these patients are immunocompromised. In addition, these patients are more likely to be elderly, and to have associated high-risk co-morbidities and behaviours.^2^ There is no published evidence to date on the post-operative and ward management of this group of patients with laboratory-proven SARS-CoV-2 infection.^3^

Here, we present our experience of the diagnosis, investigation and management of SARS-CoV-2 positive, post-laryngectomy patients, who attended our institution between 1 March and 1 May 2020, during the Covid-19 pandemic.

## Search strategy

A literature search was performed using the PubMed database. The following search criteria were used: ‘Coronavirus’, ‘COVID-19’, ‘SARS-CoV-2’, ‘laryngectomy’ and ‘head and neck carcinoma’. These searches were performed with the following limits: studies on humans, published within the last 20 years.

## Case reports

### Patient one

Patient one, a 50-year-old female, was an in-patient on the ENT ward, who was progressing well 2 weeks following total laryngectomy, bilateral neck dissection and pectoralis major flap reconstruction for a tumour–node staged T_3_N_2c_ moderately differentiated supraglottic SCC. Her past medical history included an out-of-hospital cardiac arrest and implanted cardiac defibrillator, chronic obstructive pulmonary disease (COPD), obesity, and hypertension. The patient was a lifelong smoker, although she stopped following the cardiac arrest.

Her post-operative issues included partial right neck wound dehiscence; otherwise, she was making good progress with feeding and stoma care.

Two weeks post-operatively, nursing staff highlighted the patient's frequent use of nebulisers. With clinical suspicion high because of a recently diagnosed positive healthcare worker, patient one was swab-tested and found to be positive for SARS-CoV-2. She was isolated on the infectious diseases ward, which was operating as the sole Covid-19 ward within our institution at that time. Inflammatory markers were unremarkable and there were no changes noted on chest X-ray.

Subsequent SARS-CoV-2 tests were negative and the patient returned to the ENT ward after 3 days. She remained asymptomatic throughout the remainder of her admission and was discharged home one week following her first negative test.

### Patient two

Patient two, a 66-year-old female, was an in-patient on the ENT ward following total laryngectomy, bilateral neck dissection and left hemi-thyroidectomy for a T_3_N_0_ moderately differentiated supraglottic SCC. She remained in hospital, as she developed immediate post-operative pneumonia requiring intensive care unit admission and then suffered from left neck wound dehiscence. Her past medical history included hypertension, COPD and cognitive decline, and she was an ex-smoker.

Eight weeks post-operatively, the patient developed shortness of breath, nausea and vomiting, in addition to diarrhoea. There were no recorded episodes of pyrexia; however, nursing staff did witness several episodes of rigor. Patient two was tested for SARS-CoV-2 infection and found to be positive. She was then transferred to a dedicated Covid-19 ward for ongoing care. The patient's C-reactive protein (CRP) level peaked at 109 mg/l and she developed lymphocytopenia of 0.3–0.9 × 10^9^/l. Chest X-ray demonstrated bilateral peripheral infiltrative appearances. She required 4 days of supplemental oxygen therapy before weaning, regular saline nebulisers, and nursing aid with stoma care.

The patient first tested negative for SARS-CoV-2 two weeks later. She was transferred back to the ENT ward and subsequently discharged home.

### Patient three

This patient, a 57-year-old male, was admitted to the ENT ward with symptoms of shortness of breath, increased stoma secretions and pyrexia. In 2018, patient three had undergone total laryngectomy, partial pharyngectomy and tongue base excision with bilateral neck dissection, and pectoralis major flap reconstruction, for a T_4a_N_0_ moderately to poorly differentiated supraglottic SCC. Past medical history included stroke, COPD and hypertension. He was an ex-smoker.

On admission, patient three had clinical tracheitis; however, given his symptoms, he underwent SARS-CoV-2 testing and was admitted to one of two newly formed wards for suspected or confirmed Covid-19. His admission inflammatory markers demonstrated an elevated CRP level of 39 mg/l. Chest X-ray demonstrated some alveolar shadowing within the left lower lobe. The patient was commenced on supplemental oxygen therapy, regular nebulised saline, intravenous co-amoxiclav and paracetamol. He required nursing aid with regular stoma suctioning. The following morning, his SARS-CoV-2 test returned positive. He remained isolated on the ward.

The patient made an uncomplicated clinical recovery; he was weaned from oxygen and discharged home 4 days following admission to complete a 14-day period of self-isolation.

### Patient four

Patient four, a 56-year-old female, was admitted to hospital with suspected SARS-CoV-2 infection after experiencing 2 days of cough, increased sputum production, fever and headache at home. She had previously undergone a retrograde total laryngectomy, tongue base glossectomy, bilateral neck dissection and pectoralis major flap reconstruction in 2017 for a T_4_N_0_ poorly differentiated vallecula SCC. Her other past medical history included tuberculosis and she was an ex-smoker.

Patient four was pyrexial on admission, and was therefore swab-tested for SARS-CoV-2 and transferred to a dedicated Covid-19 ward. Her test result was positive the following day. Her CRP level was 65 mg/l and there were no abnormalities on full blood count. She did not undergo a chest X-ray. She was managed with regular nebulised saline, nursing aid with stoma suctioning, and paracetamol. She did not require any supplemental oxygen therapy.

Patient four made a good clinical recovery. Her first negative test result was returned 3 days following admission and she was discharged home from hospital on the same day.

## Discussion

Evidence gathered during the pandemic consistently shows that the clinical presentation of Covid-19 includes the primary symptoms of fever, cough, and loss of taste and smell, with associated fatigue, sputum production, shortness of breath, sore throat and headache. A small proportion of Covid-19 cases suffer from gastrointestinal symptoms such as diarrhoea, nausea and vomiting. A combination of the above symptoms was exhibited in our laryngectomy patients.

The elderly, and those with medical co-morbidities such as hypertension, diabetes, COPD, cardiovascular disease and obesity, are more likely to develop severe illness such as acute respiratory distress syndrome, multi-organ failure and death.^4^ Given the pathogenesis of head and neck SCC, many of these patients who have undergone laryngectomy are highly likely to be affected by the above risk factors, which are associated with an increased risk of severe Covid-19. The age range of our patient cohort is 50–66 years; the majority of patients suffering severe disease appear to be over the age of 70 years.^2^ All patients included within our series have underlying chest disease, all are ex-smokers, three of four have hypertension and underlying cardiovascular disease, and one patient is clinically obese.

Studies have also shown associations between increased CRP levels, the presence of lymphocytopenia and severity of disease. A recent study in Wuhan demonstrated that those admitted to the intensive care unit in a severe or critical condition demonstrated lymphocytopenia (84 per cent) and an elevated CRP level (94 per cent) on admission. In addition, 76 per cent of those admitted to the intensive care unit demonstrated bilateral infiltrative changes on chest X-ray.^5^

All of our post-laryngectomy patients were managed successfully on dedicated Covid-19 wards and did not require admission to the intensive care unit. Our most severely affected patient was the eldest, patient two; she demonstrated lymphocytopenia, elevated CRP levels and bilateral infiltrative changes on chest X-ray. However, she fully recovered following two weeks of ward-based care that included oxygen therapy, saline nebulisers and regular stoma care.

The transmission of SARS-CoV-2 infection is primarily thought to spread through the respiratory tract by droplets, respiratory secretions and direct contact.^6^ Evidence of the virus has also been found in faecal and blood swabs, suggesting other routes of transmission. However, angiotensin-converting enzyme 2, the primary receptor binding site for the virus, presents in greatest concentrations in lung alveolar epithelial cells and enterocytes of the small intestine.^7^

Our experience of Covid-19 and the safe management of these patients without the requirement for a higher level of care raises the question as to whether laryngectomy may have a protective effect. We hypothesise that the lack of a direct physical route from the nasopharynx to the lungs may result in a reduction of lung viral load, causing only mild illness in an otherwise well recognised high-risk group. Further study of many more infected patients who have undergone laryngectomy is required before we can confirm or reject this hypothesis.

- Individuals who have undergone laryngectomy for head and neck squamous cell carcinoma are high risk for severe acute respiratory syndrome coronavirus-2 (SARS-CoV-2) infection
- This study focuses on post-laryngectomy patients admitted with SARS-CoV-2 infection during the outbreak peak (1 March to 1 May 2020)
- The patients responded to conservative management and were discharged without the need for higher level care
- Laryngectomy may offer protection from severe or critical coronavirus disease 2019 because of the absence of a nasopharyngeal route to the lungs

## Conclusion

Severe acute respiratory syndrome coronavirus-2 is the greatest threat to global health in recent history, and we are learning new information daily on how to best care for our patients and keep ourselves safe whilst doing so. We hope that by sharing our experience in the management of SARS-CoV-2 positive, post-laryngectomy patients, practitioners from all specialties can feel better equipped to manage this, often intimidating, group of patients safely through the Covid-19 pandemic.
